# Artificial intelligence and bioinformatics analyze markers of children's transcriptional genome to predict autism spectrum disorder

**DOI:** 10.3389/fneur.2023.1203375

**Published:** 2023-07-17

**Authors:** Huitao Tang, Jiawei Liang, Keping Chai, Huaqian Gu, Weiping Ye, Panlong Cao, Shufang Chen, Daojiang Shen

**Affiliations:** ^1^Department of Pediatrics, Zhejiang Hospital, Hangzhou, China; ^2^College of Life Science and Technology, Huazhong University of Science and Technology, Wuhan, China

**Keywords:** autistic spectrum disorder, biomarkers, RNA-Seq, neural network, LASSO

## Abstract

**Introduction:**

Autism spectrum disorder (ASD), characterized by difficulties in social interaction and communication as well as restricted interests and repetitive behaviors, is extremely challenging to diagnose in toddlers. Early diagnosis and intervention are crucial however.

**Methods:**

In this study, we developed a machine learning classification model based on mRNA expression data from the peripheral blood of 128 toddlers with ASD and 126 controls. Differentially expressed genes (DEGs) between ASD and controls were identified.

**Results:**

We identified genes such as UBE4B, SPATA2 and RBM3 as DEGs, mainly involved in immune-related pathways. 21 genes were screened as key biomarkers using LASSO regression, yielding an accuracy of 86%. A neural network model based on these 21 genes achieved an AUC of 0.88.

**Discussion:**

Our findings suggest that the identified neurotransmitters and 21 immune-related biomarkers may facilitate the early diagnosis of ASD. The mRNA expression profile sheds light on the biological underpinnings of ASD in toddlers and potential biomarkers for early identification. Nevertheless, larger samples are needed to validate these biomarkers.

## 1. Introduction

Autism spectrum disorder (ASD) is a neurodevelopmental disorder characterized by the impairment of social and communication skills and repetitive movements in early childhood (1). Genetic factors are associated with the susceptibility to and development of ASD, with an estimated heritability of 50–83% (2). To date, the pathological mechanisms and curative treatment of ASD have not been clarified (3, 4). Early diagnosis and interventions could significantly improve the life of ASD toddlers (5). Reportedly, ASD is difficult to diagnose in toddlers under 53 months of age (6).

The current methods are either expensive or subjective, and their application in the diagnosis of ASD is limited. Research on the use of electroencephalography (EEG) data from children with ASD to train neural networks for predicting ASD has yielded promising results. However, EEG signals are susceptible to ambient noise, and there may be significant individual differences, which pose practical limitations in their application (7). Similarly, studies utilizing infant functional magnetic resonance imaging (fMRI) data and machine learning methods have shown potential for the classification of ASD. Nevertheless, fMRI scanning can be challenging for infants who require a calm and still environment, leading to poor image quality and motion artifacts (8). Additionally, genomic data analysis has identified genetic risk variants associated with ASD. However, single gene mutations alone are not sufficient for fully explaining the complexity of ASD (9). Therefore, further research is necessary to explore the potential of these approaches in accurately predicting ASD in children.

Identifying ASD biomarkers may help with the early diagnosis of ASD. However, the well-known neural system biomarkers of ASD are rarely applied due to the difficulty of sample collection. Compared to neural system tissue, peripheral blood is more readily available to screen for biomarkers, but identifying blood biomarkers for ASD and using them to diagnose ASD are two core issues that need to be resolved. The impetus herein was interrogating mRNA expression profiles of peripheral blood from ASD subjects and controls to obtain biomarkers that are amenable to ASD diagnostics. Expression microarray data from 128 ASD and 126 control toddlers were analyzed. Differential expression analysis revealed that 1,027 genes (adjusted *P* < 0.05) were dysregulated in ASD; the ingenuity pathway analysis of the top 200 genes identified immune response, neurotransmission, and cell proliferation pathways as enriched. The least absolute shrinkage and selection operator (LASSO) regression identified 21 candidate biomarkers, including *GDI1, HYAL3*, and *ANAPC7*. Binary logistic regression and neural network models were developed utilizing these 21 biomarkers, achieving a satisfactory accuracy of 86 and 88%, respectively. These models demonstrated the potential of the identified biomarkers for the early detection of ASD.

In aggregate, we adduced 21 candidate peripheral blood biomarkers related to immune functions, growth factors, and neurotransmitter functions in ASD. Our methodology and results adumbrate the value of biomolecular approaches coupled with machine learning for illuminating pathological mechanisms underlying ASD and developing diagnostic modalities. Although the findings are promising, further validation in larger, heterogeneous populations and comparison with prevailing diagnostics are necessary to determine their clinical utility.

## 2. Methods

### 2.1. Data acquisition and preprocessing

The data used in this study were obtained from the Gene Expression Omnibus (GEO) database in NCBI (Gene Expression Omnibus, http://www.ncbi.nlm.gov/geo), and the access number is GSE111175 (10, 11), GSE42133 (12, 13). The platform is Illumina HumanHT-12 V4.0 Expression BeadChip. Gene expression data of 128 ASD and 126 CON samples were identified. In addition, 38 developmental delay (27 language delay samples, 9 pervasive developmental disorder not otherwise specified samples, 1 socially and emotionally delayed sample, 1 global developmental delay sample) samples in GSE111175 were identified for specificity detection of the machine learning model. Principal component analysis (PCA) was performed to visualize the batch effect between the two datasets. The batch effect was eliminated using the SVA package based on the R language (14). Expression matrix probes without corresponding annotation were removed. Finally, we obtained normalized and batch effect-removed RNA expression data, which contained 254 samples and 24,698 genes.

### 2.2. Diagnostic criteria and procedures for ASD

The diagnosis of ASD was determined using the datasets GSE111175 and GSE42133, which also provide the age, the Autism Diagnostic Observation Schedule (Module T, 1, or 2) (ADOS) scores (15), the Mullen Scales of Early Learning (MSEL) (16) scores of the ASD and CON samples, and the Vineland Adaptive Behavior Scales (17) scores of the ASD and CON samples. A *t*-test was performed to calculate the *p-*values of scores between the two groups.

### 2.3. Clustering methods

A cohort of 128 toddlers diagnosed with ASD was assessed using a dataset consisting of 12 clinical features, including scores from the ADOS, MSEL, and Vineland. In particular, ADOS scores encompassed communication, sociability, circumscribed, and repetitious behaviors, while MSEL scores encompassed precocious learning aggregate and fine motor skills. The Vineland scores encompassed activities of daily living and motor adaptive functioning.

To analyze the data, both hierarchical and K-means clustering methods were employed, with the number of clusters set to two for each technique. The K-means algorithm utilizes a random state of 42. By calculating the spatial distances among the 12 indices across the entire cohort, the toddlers were effectively stratified into two distinct clusters, providing valuable insights into the heterogeneity of ASD profiles.

### 2.4. Identification of differential expression genes

The Wilcox test in the R-package “limma” was performed to screen for differentially expressed genes (DEGs) (18). The Benjamini–Hochberg method, which is a multiple testing method, was performed to calculate the adjusted *p*-values and reduce false-positive DEGs. The heatmap and scatter plot of DEGs were plotted using the R language packages “pheatmap” and “ggpubr.” A total of 200 DEGs with the largest absolute value of LogFC were used as the top 200 candidates for subsequent analysis.

### 2.5. Functional enrichment analysis of DEGs

The Gene Ontology enrichment analysis of DEGs was based on R language packages “org.Hs.eg.db” and “enrichplot.” The gene set enrichment analysis (GSEA) function enrichment analysis was performed using the R language package “ReactomePA” (19, 20). The number of permutations was set to 100, and a *p*-value of <0.05 and an FDR of <0.25 were considered statistically significant. Ingenuity pathway analysis (IPA) was conducted using IPA software with input data for the gene symbol and the logFC value of DEGs with an adjusted *p*-value of <0.05.

### 2.6. The construction of machine learning models

The LASSO regression model, which is suitable for dimensionality reduction of high-dimensional data, was used to screen genes with non-zero coefficients. The expression data of the screened genes were used to train the logistic regression model and construct the nomograph. The glmnet (21), rms, and regplot (22) packages based on the R language were used to implement the above method. The expression data of the screened genes were used to train and optimize the neural network model using the sklearn package in Python.

### 2.7. The acquisition and pre-processing of the test dataset

The test dataset was GSE26415 (23). The platform was Agilent-014850 Whole Human Genome Microarray 4x44K G4112F. The original datasets contained four groups of 21 toddlers each: toddlers with ASDs, healthy age- and sex-matched subjects (ASD and CON, ages ranging from 18 to 35 months), healthy mothers who had toddlers with ASD (ASDmos), and CONmos (ages ranging from 33 to 55 months). Gene expression data of the 21 ASD and 21 CON participants were retained. The normalized data were downloaded, and the expression matrix was obtained.

### 2.8. Validation of ASD early diagnosis nomogram

A calibration curve method was adopted to evaluate the coordination between the predicted and actual ASD. A decision curve was adopted to quantify the net benefits of different threshold probabilities in the ASD cohort and evaluate the clinical utility of the strong line chart. The net benefits were calculated by subtracting the proportion of all false positive patients from the proportion of true positive patients. The relative harm caused by the intervention was balanced, and the negative consequences of unnecessary interventions were eliminated. The visualization of the decision curve was based on the RMDA package of the R language. The generalization ability and accuracy of the trained model were evaluated using the test datasets.

## 3. Results

### 3.1. Identification and removal of batch effects and normalization of datasets

We identified an obvious batch effect between the two datasets (GSE111175 and GSE42133) via PCA (Supplementary Figure S1A), which could be removed via the SVA package (Supplementary Figure S1B). To eliminate the within-group inconsistency and normalize the dataset, limma packages were used (Supplementary Figures S1C, D).

### 3.2. The analysis of participant characteristics and clinical information

**Figure 1:**
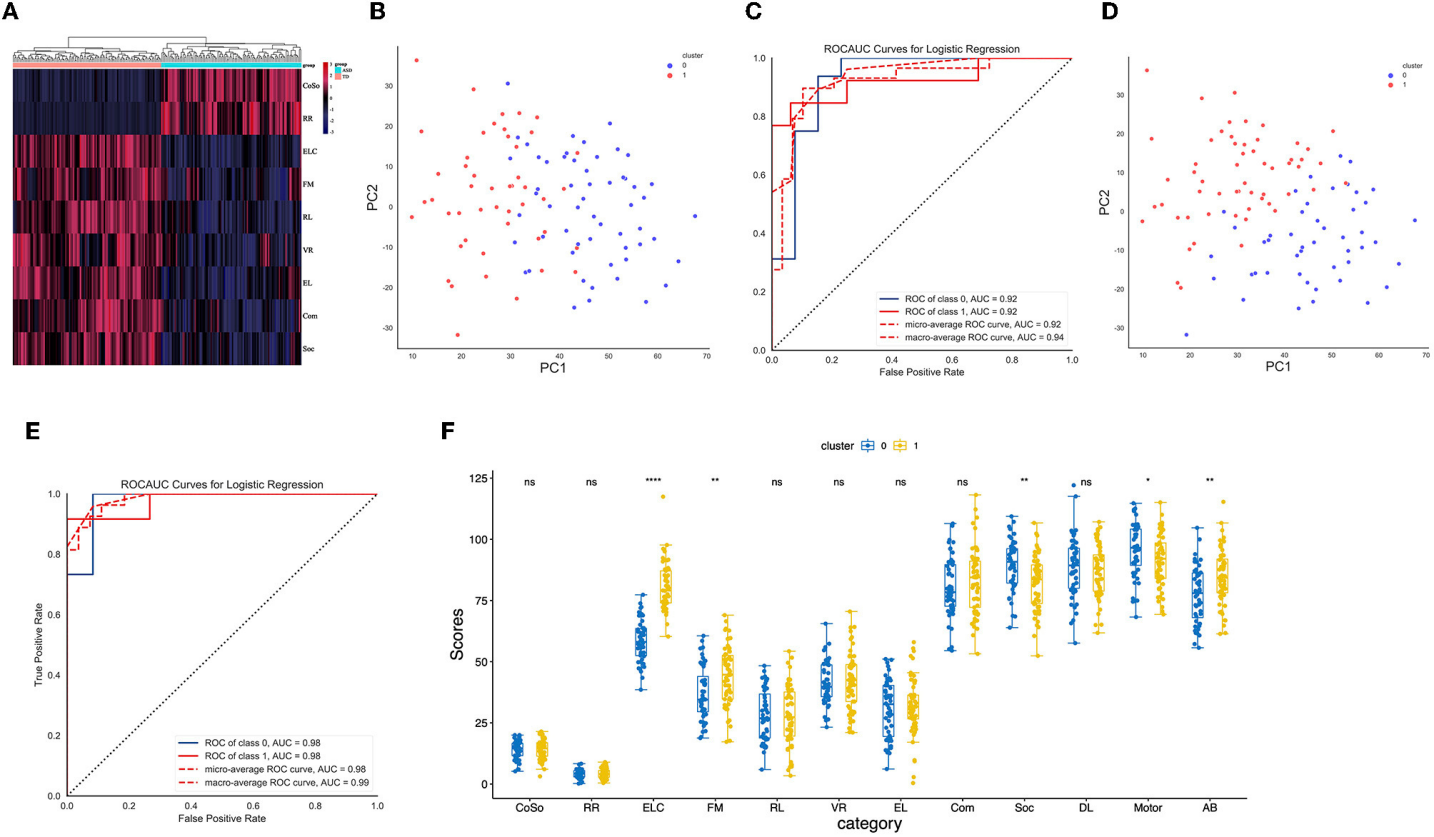
**(A)** A heatmap of the clinical feature score for CON and ASD samples. **(B)** Distribution of hierarchical clusters 0 and 1 of ASD in PC1 and PC2 spaces. **(C)** Logistic regression identifies the receiver-operating characteristic (ROC) curves of hierarchical ASD-0 and ASD-1 **(D)** Distribution of ASD-0 and ASD-1 clusters in PC1 and PC2 spaces identified by k-means. **(E)** Logistic regression identifies the ROC curves of k-means ASD-0 and ASD-1. **(F)** The boxplot shows the difference in clinical feature scores between ASD-0 and ASD-1.

First, differences in the clinical scores between the ASD and CON participants were identified (Supplementary Table S1, Figure 1A). Specifically, we compared the ADOS scores of the ASD and CON participants and observed that the ASD participants had significantly higher ADOS scores than the CON participants. Similarly, MSEL score differences between the two groups were identified. Furthermore, we also observed that the Vineland scores of the ASD participants were lower than those of the CON participants.

To assess whether there was a within-group bias within the ASD cohort, such as the presence of a good or poor ASD group, hierarchical cluster methods were employed. In particular, 61 ASD toddlers in cluster 0 and 67 ASD toddlers in cluster 1 were identified (Figure 1B). The confidence of the clusters was verified by logistic regression (AUC = 0.92, Figure 1C). To improve the accuracy of clustering, the k-means clustering method was employed. Ultimately, 66 ASD toddlers in cluster 0 and 62 ASD toddlers in cluster 1 were identified (Figure 1D). The confidence of the clusters was verified by logistic regression (AUC = 0.98, Figure 1E). Furthermore, we identified that early learning composite (ELC) and adaptive behavior (AB) scores in ASD cluster 1 were higher than those in ASD cluster 0 (Figure 1F).

### 3.3. Identification of DEGs between ASD and CON, and the functions of DEGs

**Figure 2:**
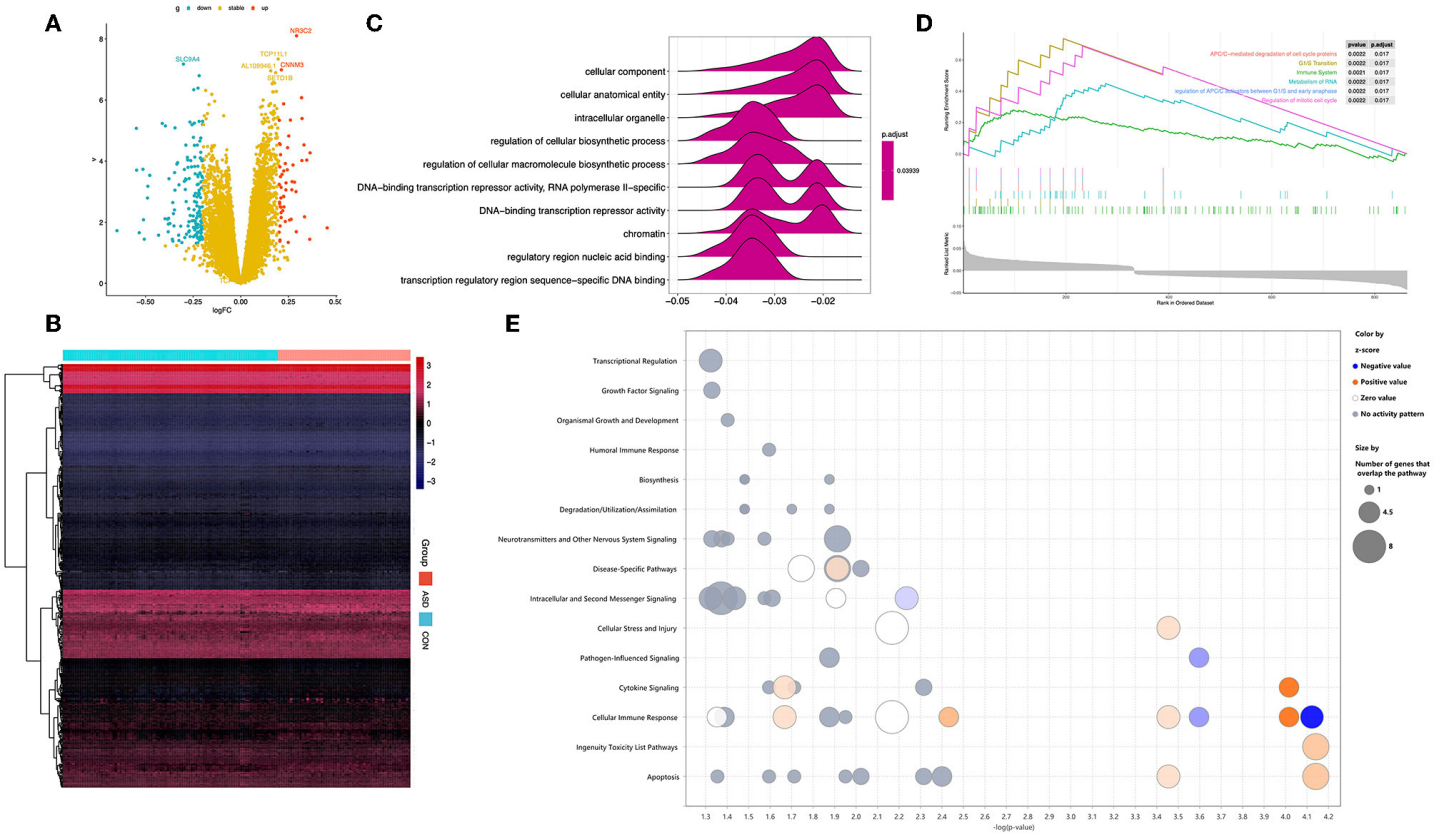
Identification of DEGs between ASD and CON and DEGs functional enrichment analysis. **(A)** Volcano diagram: Each point represents a protein: downregulated (blue), upregulated (red), and not significant (yellow). **(B)** A heatmap of the expression of DEGs in ASD and CON. **(C)** TOP-6 GSEA analysis of the KEGG pathway in DEGs. **(D)** Statistically significant Gene Ontology terms with a false discovery rate of <0.05. **(E)** The canonical pathway of IPA shows the signaling pathways in which DEGs are involved.

We identified 1,027 DEGs between 128 ASD and 126 CON participants based on the pre-processing expression data (Figures 2A, B). GO analysis was performed to identify the function of these DEGs; regulatory region nucleic acid binding and transcription regulatory region sequence-specific DNA binding were identified as the main molecular functions. Furthermore, the results of GSEA indicated that the main function of DEGs was APC-mediated degradation of cell cycle proteins, the immune system, and so on (Figures 2C, D). We also analyzed the canonical pathways of DEGs through IPA and found that the functions of the DEGs are enriched in immune-related signaling pathways such as cellular immune response and cytokine signaling. In addition, growth-related signaling pathways, neurotransmitter pathways, and other nervous signaling pathways were identified (Figure 2E).

### 3.4. Construction of nomogram for the early diagnosis of ASD

**Figure 3:**
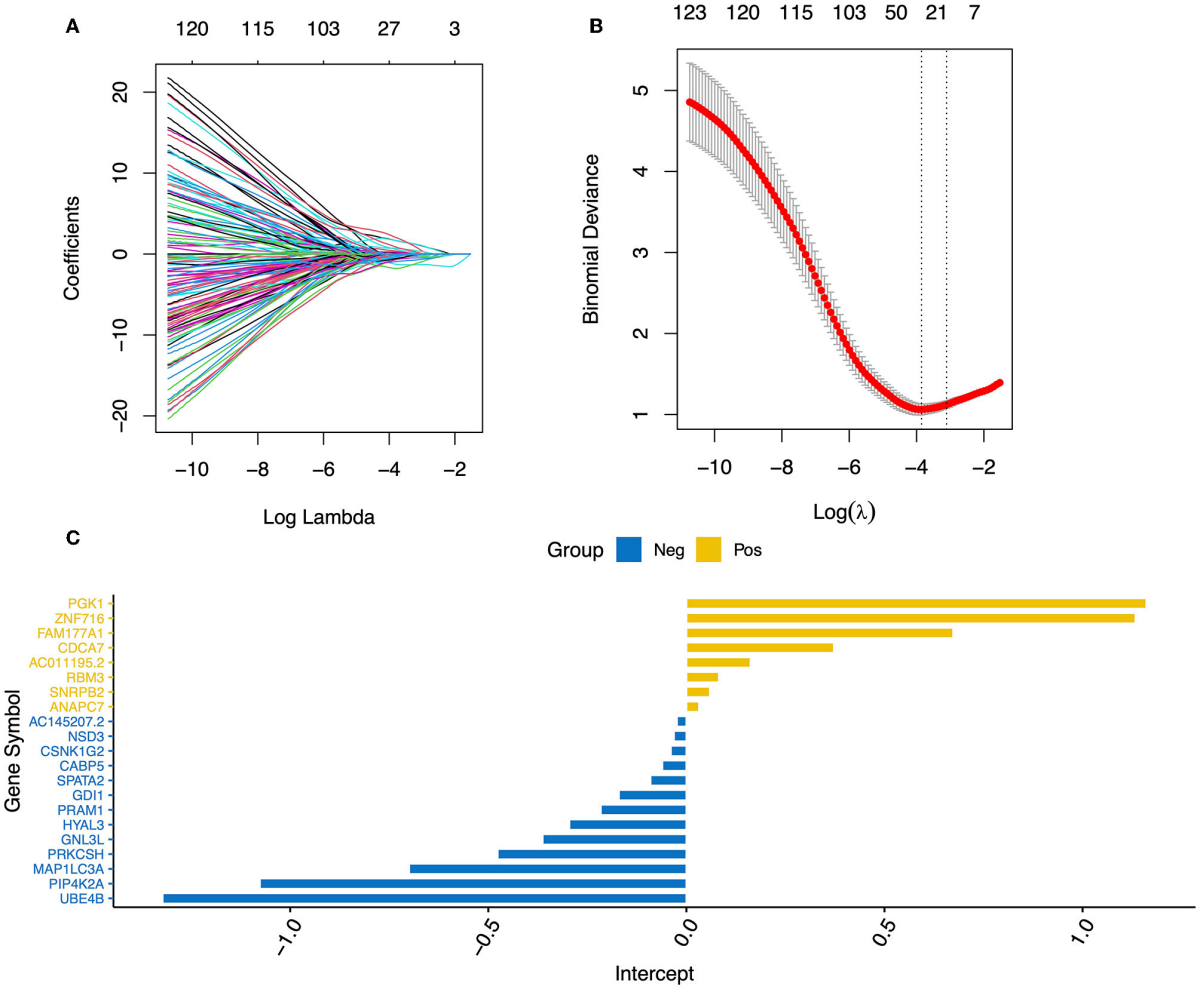
Blood feature gene selection using the LASSO binary logistic regression model. **(A)** LASSO coefficient profiles of the 200 DEGs. **(B)** A coefficient profile plot was produced against the log (lambda) sequence in the LASSO model. The optimal parameter (lambda) was selected as the first black dotted line indicated. **(C)** A bar graph of the coefficients of the 21 genes selected.

**Figure 4:**
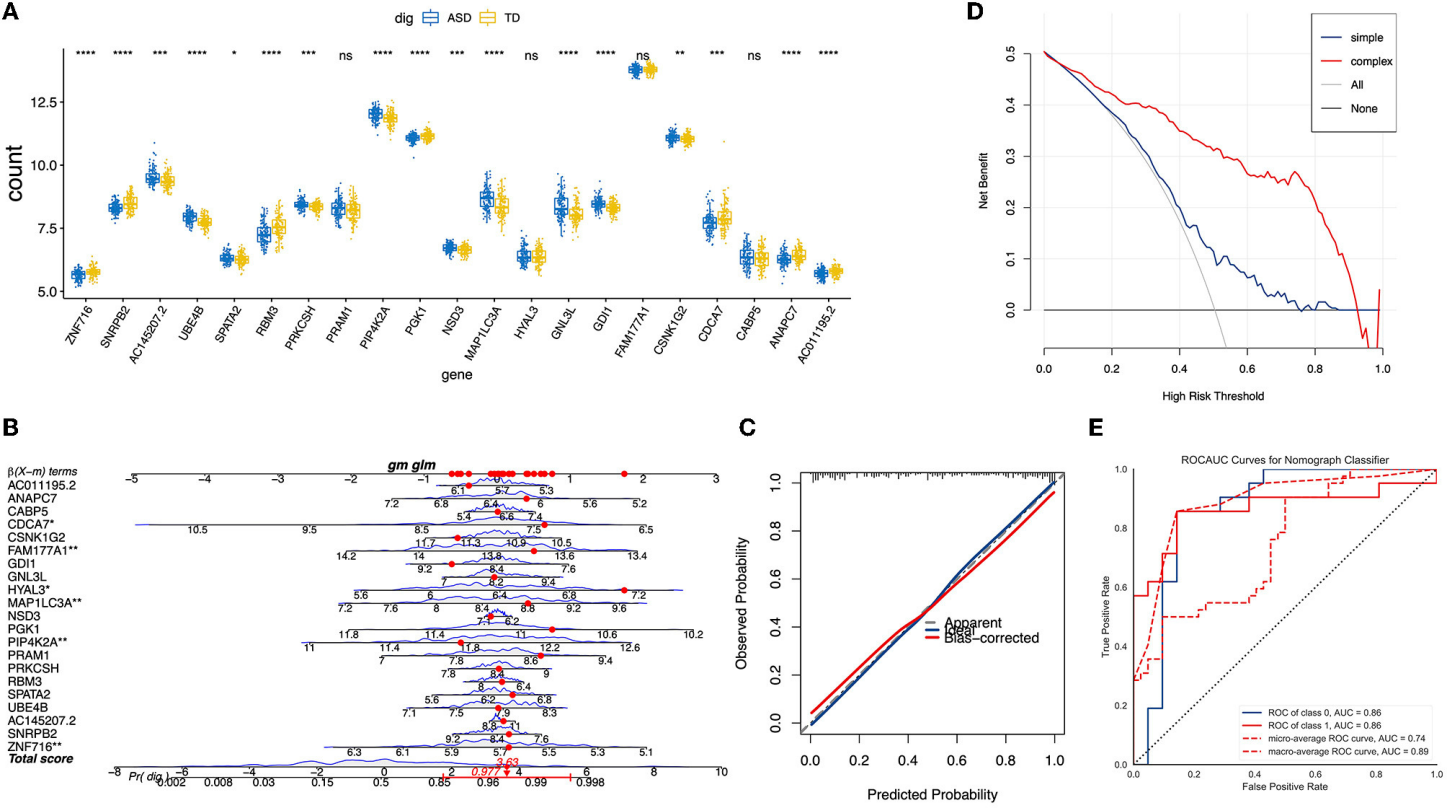
Construction and validation of ASD diagnosis nomogram. **(A)** A boxplot of the expression values of the 21 genes selected in different samples. **(B)** The nomogram for predicting the risk of ASD based on 21 genes. **(C)** The calibration curves of the ASD prediction nomogram. **(D)** The ROC curve of the nomogram in the validation cohort. **(E)** The decision curve analysis for the nomogram.

To limit the overfitting of machine learning models and screen the DEGs, LASSO regression was performed. Specifically, we selected 200 genes with large logFC absolute values from 1,027 DEGs for LASSO regression, and 21 DEGs were retained (Figures 3A–C). These genes included *ZNF716, SNRPB2, AC145207.2, UBE4B, SPATA2, RBM3, PRKCSH, PRAM1, PIP4K2A, PGK1, NSD3, MAP1LC3A, HYAL3, GNL3*, and so on. Logistic regression analysis and nomography were performed to construct a diagnosis model of ASD based on the expression data of the 21 DEGs (Figures 4A, B). In addition, the calibration curve demonstrated the consistency between the predictions of the nomograph and the cohort (Figure 4C).

To evaluate the generalization ability of the logistic regression model and the benefits of the nomogram in clinical use, we assessed the accuracy of prediction of the trained logistic regression model in the test dataset, as shown in Figure 4D, AUC = 0.86. Furthermore, the decision curve of the nomogram indicated a higher net benefit of using the complex model than using the single gene model (Figure 4E).

### 3.5. Construction of the artificial neural network model

**Figure 5:**
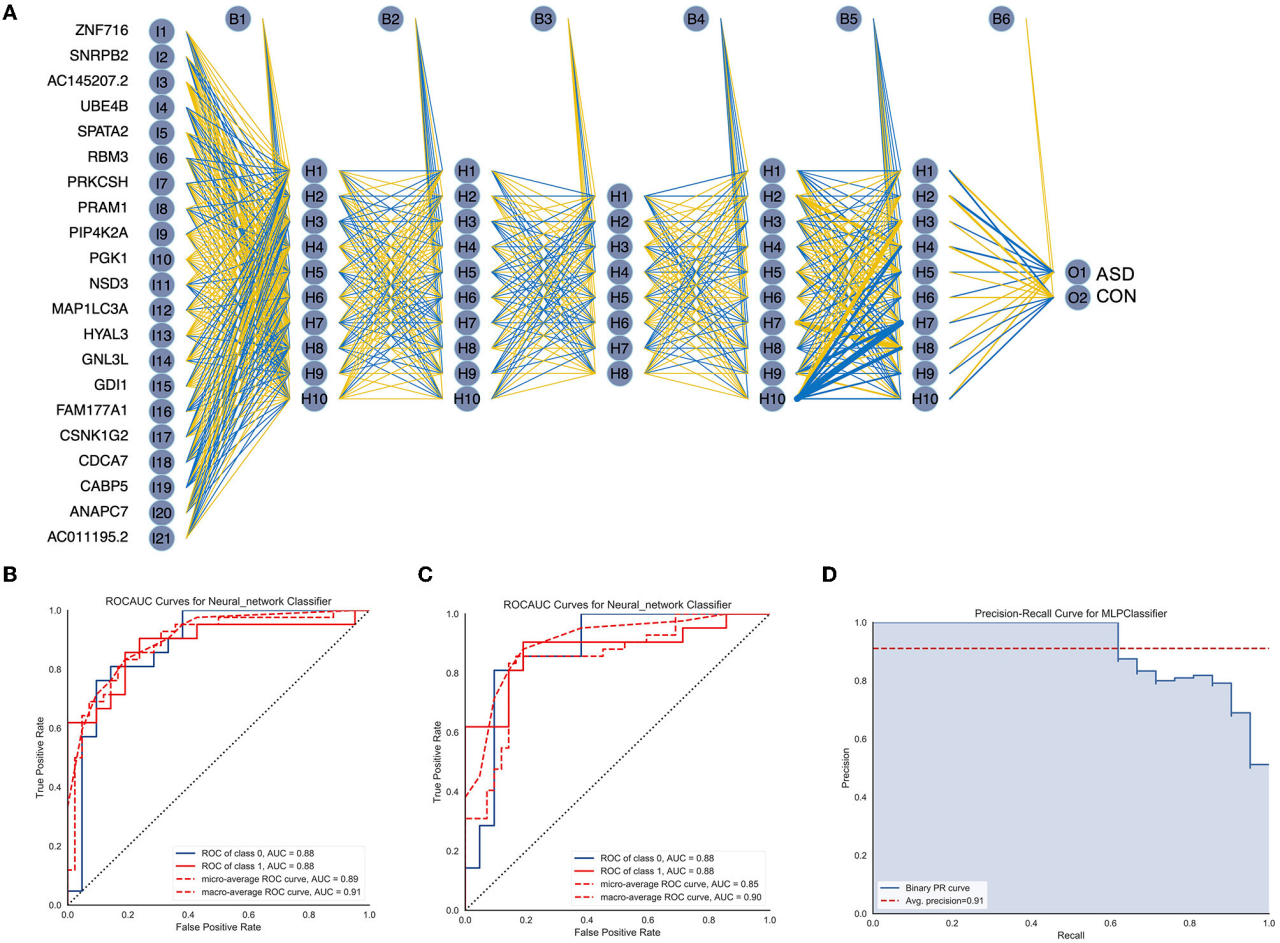
Construction and validation of the ASD diagnosis neural network. **(A)** Results of neural network visualization. **(B)** The ROC curve of the neural network in the training cohort. **(C)** The ROC curve of the neural network in the validation cohort. **(D)** The precision-recall curve for the neural network classifier.

To further improve the accuracy of ASD diagnosis, we used the Python-based sklearn package to construct a neural network model that could predict ASD. Specifically, the above 21 gene expression matrix was used to train the neural network model, which had five hidden layers, and the number of neurons included in each hidden layer was 10, 10, 8, 10, and 10, as shown in Figure 5A. The number of iterations was set to 200, ReLU was selected as the activation function, the weight optimizer was the optimizer of the quasi-Newton method, and the regularization parameter was 0.00001. The average AUC of the model in the training dataset was 0.88, as shown in Figure 5B. Next, we evaluated the generalization ability of the trained neural network model in the test dataset (n ASD = 21, n CON = 21), as shown in Figure 5C, AUC = 0.88. The precision-recall curve (Figure 5D, average precision = 0.91) indicated that the trained neural network had improved generalization and accuracy.

In addition, to prevent the differences within the ASD dataset from affecting the prediction results of the model, the data of ASD good and ASD poor were used to construct the neural network model. Firstly, 66 ASD good and 126 CON samples were used to construct the neural network model of the above structure, and the accuracy of the model in predicting the ASD good and CON samples was 0.87 through 5-fold cross-validation (Supplementary Figure S2A). Similarly, we also built the neural network model of ASD poor and CON samples and found that the accuracy of the model was 0.85 (Supplementary Figure S2B). To explore the 21 genes' ability to distinguish between ASD good and poor samples, 66 ASD good and 62 ASD poor samples were used to construct the neural network, and we found that the accuracy of this model was 0.68 (Supplementary Figure S2C). Finally, to explore the specificity of the neural network model in predicting ASDs, 38 toddlers with developmental delays were considered in the analysis, and the accuracy of the model in predicting 38 developmental delays was 0.52 (Supplementary Figure S2D).

## 4. Discussion

Early diagnosis and targeted interventions for ASD in toddlers are imperative to optimize neurodevelopmental outcomes and quality of life (5); yet, current diagnostic methods are subjective, cost-prohibitive, and constrained by developmental maturation. The current diagnosis of ASD includes multiple dimensions, such as vision and hearing examination to exclude sensory impairment, genetic and neurological testing, interviews with parents, neurological or psychiatrist observation of children, and development and behavioral tests of children (24–26). These special evaluation or inspection methods are either subjective or expensive. In addition, the cognitive assessment for toddlers may vary depending on their age. Thus, there is an urgent need to develop a safe, convenient, and accurate method for ASD diagnosis.

Biomarker-based machine learning models could enable scalable, accurate, and objective early detection of ASD, which is critical to transforming prognosis. Compared with other methods, biomarkers are more objective and convenient (27, 28). With the development of machine learning algorithms, the accuracy of diagnosis can be improved by combining machine learning methods with biomarker data. However, two issues remain and must be resolved. First, machine learning usually requires a large amount of data (29, 30). To expand the dataset, datasets GSE111175 and GSE42133 from the GEO database, which included 128 ASD participants and 126 CON participants in the datasets, were obtained. Second, limiting the features of the data to avoid overfitting the model and improving the generalization ability of the model are necessary. In this study, several methods were employed to reduce the dataset's dimensions, such as selecting the top 200 genes from 1,027 DEGs (Figures 2A, B) and LASSO regression (Figure 3). In addition, 21 key DEGs were identified as the biomarkers to diagnose ASD (Figures 3, 4A). To evaluate the generalization ability of the model, we compared the predicted results of the trained dataset with the predicted results of the test dataset (GSE26415, 21 CON, 21 ASD) and found that the difference in accuracy between the two was negligible, indicating that there was no serious overfitting in the trained model (Figure 4D). We also found that the net benefit of using the complex model was higher than the benefit of using a single gene model, indicating the strong robustness of the trained model (Figure 4E). To obtain a more accurate model, the neural network model was also trained in this study (Figure 5A). Similarly, we found that the accuracy of the neural network in the test data was higher than that of the nomograph (AUC = 0.88, Figures 5B, C). Although the improvement in the accuracy of the model was not as obvious as in other studies (13, 23), we established the robustness of the model across different datasets. To prevent the interference of intra-group differences in ASDs with the prediction results, we redefined neural network models as ASD good and ASD poor, respectively. Compared with the original neural network model, the accuracy of the ASD good and ASD poor models decreased, which may have been caused by the reduction of the dataset size (Supplementary Figures S2A, B). In addition, we found that the neural network model used to distinguish ASD good from ASD poor did not show high accuracy, which may have been due to the insignificant difference in biomarkers between the two groups (Supplementary Figure S2C).

Many previous studies have identified biomarkers related to the immune system in blood samples from individuals with ASD (13, 31–33), which is consistent with our results that the DEG pathways are mainly distributed in the immune system (Figures 2C–E). Furthermore, the growth factor, organismal growth and development, neurotransmitters, and other nervous system signaling were established through IPA analysis, which may indicate the novel and specific pathways related to ASD biomarkers (Figure 2E). Among these 21 biomarkers, we identified that *GDI1* expression in ASD was significantly increased (Figure 4A), which can be explained by the recurrent duplications of *GDI1* in ASD (34). In a previous study, *GDI1*-related pathways were reported to be a vesicle-mediated transport (35), which may be related to the neurotransmitter pathway identified in the IPA (Figure 2E). A genome-wide association study analysis showed that *HYAL3* is one of the pathogenic genes of attention-deficit hyperactivity disorder (36). In our study, the expression of *HYAL3* in ASD was significantly increased (Figure 4A), indicating that *HYAL3* may also cause ASD. Similarly, a decrease in *ANAPC7* expression in ASD was also identified (Figure 4A). Studies have shown that the loss of *ANAPC7* is associated with intellectual disability syndrome (37), which may also explain the intellectual disability in toddlers with ASD.

The final limitation of our study is the generalization ability of the neural network model, which can be better assessed with the help of multiple test datasets. We encountered difficulty in finding the expression profile data of blood leukocyte samples for ASD in open databases, which prevented us from verifying the generalizability of the model in this study. Thus, in a future study, we will explore whether specific data are available for this purpose.

## 5. Conclusion

In this study, we obtained blood RNA-seq data of CON and ASD toddlers from public GEO datasets in NCBI and identified the potential biomarkers of ASD, including ANAPC7 and HYAL3, through a series of analyses. Machine learning can be used on the expression data of the biomarkers to obtain models with high prediction accuracy. By predicting different datasets, we established a certain level of generalizability of our model. We also predicted the developmental delay dataset and established that our model has a certain specificity. Further improvements to the model may shed some light on its clinical application.

## Data availability statement

The datasets presented in this study can be found in online repositories. The names of the repository/repositories and accession number(s) can be found below: https://www.ncbi.nlm.nih.gov/geo/query/acc.cgi?acc=GSE111175; https://www.ncbi.nlm.nih.gov/geo/query/acc.cgi?acc=GSE42133.

## Ethics statement

Ethical review and approval was not required for the study on human participants in accordance with the local legislation and institutional requirements. Written informed consent from the patients/participants or patients/participants' legal guardian/next of kin was not required to participate in this study in accordance with the national legislation and the institutional requirements.

## Author contributions

HT contributed to the study design, performed the experiments, and contributed to the writing of the manuscript. JL contributed to the data collection and writing of the manuscript. KC, HG, WY, PC, and SC contributed to the study design. DS contributed to the study design, performed the experiments, and contributed to the writing of the manuscript. All authors read and approved the final manuscript.
