# Supplementary material for: Artificial intelligence and bioinformatics analyze markers of children's transcriptional genome to predict autism spectrum disorder
Source: Front Neurol. 2023 Jul 17;14:1203375. doi: 10.3389/fneur.2023.1203375 (PMC10390071; doi:10.3389/fneur.2023.1203375)
Supplement: Supplementary file 1 [file Table_1.docx]

**

**

**Figure.S1 A,B**. Principal component analysis before(**A**) and after(**B**) removing batch effects from the merged dataset. **C,D.** Boxplot of expression matrix before(**C**) and after(**D**) normalization.

**Table.S1 Summary of Participant Characteristics and Clinical Information**

| **Variable** | | **CON** | | **ASD** |
| --- | --- | --- | --- | --- |
| **N** | 126 | | 128 | |
| **Age (Months)** | 23.5 (9.86) | | 28.4 (8.85) | |
| **ADOS** |  | |  | |
| Communication and social (COSO) | 2.65 (2.06) | | 14.0 (3.82) | |
| Restricted, repetitive (RR) | 0.49 (0.36) | | 4.21 (1.90) | |
| **MSEL** |  | |  | |
| Early learning composite (ELC) | 111 (15.31) | | 72.9 (15.92) | |
| Fine motor (FM) | 57.1 (10.54) | | 37.6 (12.53) | |
| Receptive language (EL) | 52.4 (9.89) | | 27.7 (11.82) | |
| Visual reception (VR) | 56.92 (11.00) | | 38.7 (9.94) | |
| Expressive language (EL) | 53.7 (9.55) | | 30.9 (10.90) | |
| **Vineland** |  | |  | |
| Communication (Com) | 106.66 (9.04) | | 78.96 (13.40) | |
| Socialization (Soc) | 106.23 (9.37) | | 84.21 (11.17) | |
| Daily Living (DL) | 104.02 (9.14) | | 86.25 (10.85) | |
| Motor | 100.83 (7.57) | | 91.63 (12.40) | |
| Adaptive Behavior (AB) | 105.23 (8.30) | | 82.52 (11.40) | |

**Figure.S2 A.** The ROC curve of neural network in the ASD cluster 0 and CON. **B.** The ROC curve of neural network in the ASD cluster 1 and CON. **C.** The ROC curve of neural network in the ASD cluster 0 and cluster 1. **D.** The ROC curve of neural network in the development delay samples and CON samples.
